# Dopamine D3 receptor antagonist reveals a cryptic pocket in aminergic GPCRs

**DOI:** 10.1038/s41598-018-19345-7

**Published:** 2018-01-17

**Authors:** Noelia Ferruz, Stefan Doerr, Michelle A. Vanase-Frawley, Yaozhong Zou, Xiaomin Chen, Eric S. Marr, Robin T. Nelson, Bethany L. Kormos, Travis T. Wager, Xinjun Hou, Anabella Villalobos, Simone Sciabola, Gianni De Fabritiis

**Affiliations:** 1 Computational Biophysics Laboratory (GRIB-IMIM), Universitat Pompeu Fabra, Barcelona Biomedical Research Park (PRBB), Doctor Aiguader 88, 08003 Barcelona, Spain; 2 Acellera, PRBB, Doctor Aiguader 88, 08003 Barcelona, Spain; 3 Institució Catalana de Recerca i Estudis Avançats (ICREA), Passeig Lluis Companys 23, 08010 Barcelona, Spain; 4 Pfizer Worldwide Research and Development, 1 Portland Street, Cambridge, Massachusetts 02139 United States; 5 Pfizer Worldwide Research and Development, Eastern Point Road, Groton, Connecticut 06340 United States

## Abstract

The recent increase in the number of X-ray crystal structures of G-protein coupled receptors (GPCRs) has been enabling for structure-based drug design (SBDD) efforts. These structures have revealed that GPCRs are highly dynamic macromolecules whose function is dependent on their intrinsic flexibility. Unfortunately, the use of static structures to understand ligand binding can potentially be misleading, especially in systems with an inherently high degree of conformational flexibility. Here, we show that docking a set of dopamine D3 receptor compounds into the existing eticlopride-bound dopamine D3 receptor (D3R) X-ray crystal structure resulted in poses that were not consistent with results obtained from site-directed mutagenesis experiments. We overcame the limitations of static docking by using large-scale high-throughput molecular dynamics (MD) simulations and Markov state models (MSMs) to determine an alternative pose consistent with the mutation data. The new pose maintains critical interactions observed in the D3R/eticlopride X-ray crystal structure and suggests that a cryptic pocket forms due to the shift of a highly conserved residue, F^6.52^. Our study highlights the importance of GPCR dynamics to understand ligand binding and provides new opportunities for drug discovery.

## Introduction

G-protein coupled receptors (GPCRs) are one of the largest membrane protein families, encoded by more than 800 genes^1^. GPCRs recognize a wide range of extracellular signals and transduce them into diverse cellular responses. Blocking or altering these signals provides extraordinary pharmacological opportunities; not surprisingly, ~34% of marketed drugs target GPCRs^1^. Understanding GPCR binding pocket recognition elements is thus a first step toward designing drugs that modulate these responses.

When first characterized, GPCRs were thought to operate as ‘on-off’ switches through two unique active and inactive states. Recent studies show that GPCRs are more complex than this and have characterized the pathways of entry of different GPCR ligands^2^, their conformational ensemble landscape^3^, and many activation pathways^4,5^. In addition, GPCRs adopt a set of differently populated active, inactive, and intermediate sub-states, and it has been shown that diverse ligands and coupling proteins interact with GPCRs and shift their populations to different extents^6^.

The most studied GPCR class is the rhodopsin-like class A GPCR family, divided into more than 50 subfamilies^7^. Some of the rhodopsin-like class A GPCRs recognize biogenic amines as endogenous agonists and are categorized as aminergic receptors. Up to 25% of the currently marketed drugs bind to aminergic receptors, and eleven distinct ligand-bound active and inactive crystal structures have been published^7^. Aminergic subfamilies include the muscarinic, adrenergic, histamine, trace amine, serotonin and dopamine receptors^8^.

D3 receptor (D3R) antagonism is a compelling molecular mechanism for its role in drug addiction. D3R modulation has been pursued for more than a decade as a potential target for the treatment of substance use disorders^9,10^, and several selective D3R antagonists have been reported in the literature^11–13^. Most of the D3R antagonist structures disclosed to date share a common 4-feature pharmacophore (A_1_-B-L-A_2_), where an aromatic (A_1_) group fills the orthosteric binding site (OBS) followed by a basic cyclic amine (B) hydrogen bonding to the conserved D^3.32^, connected to a flexible linear chain (L) linked to a second aromatic (A_2_) group that explores divergent interactions within the secondary binding pocket (SBP)^11–14^.

We recently published the *in vitro* and *in vivo* characterization of PF-4363467 (**1**, Table 1), a D3R dual antagonist with unusually high D2R receptor occupancy (86%), which translated into unique *in-vivo* signaling, e.g. lack of locomotor or catalepsy side effects^15^. Unlike previously reported structures of D3R antagonists, **1** does not share the same pharmacophore feature arrangement (B-A_1_-L-A_2_) and presented some challenges in the prediction of its binding mode^15^. Induced-fit docking of **1** into the published D3R X-ray structure (PDB ID 3PBL)^16^ suggested the possibility of two binding modes, neither of which was fully predictive of the SAR and selectivity in the series^15^. The true binding mode of **1** to D3R therefore remains unclear.

**Table 1:** Summary of compounds studied in this work.

ID	Name	2D structure	*K*_i_ (nM)	Mutations
**1**	PF-4363467		3.4 ± 0.4	I183^ECL2^F
				V189^5.39^A
**2**	Eticlopride		0.24^16^	V189^5.39^I
				Y373^7.43^F
**3**	Haloperidol		6.5 ± 1.0	C114^3.36^L,
				I183^ECL2^F
				E90^2.65^Q
**4**	GSK598809		2.5 ± 0.4	Y36^1.39^L
				E90^2.65^Q
				Y373^7.43^F

Structure, inhibition constant (*K*_i_), and mutations that most affected binding for each compound. Values presented were measured in this work except eticlopride (**2**), measured in ref.^16^.

In order to better understand how **1** potentially binds to the D3R and exerts its therapeutic effect, a comparative study of **1** was performed with three other well-characterized D3R antagonists: eticlopride **(2)**, which has been co-crystallized in complex with D3R, haloperidol **(3)**, the most widely used first-generation antipsychotic, and GSK598809 **(4)**, a dual D2R/D3R antagonist which has been shown to be active for the treatment of drug abuse disorder (Table 1).

Point mutation studies were performed on the D3R to identify key binding interactions for the four antagonists. Using rigid docking the binding poses of **2**, **3** and **4** could be rationalized with the point mutation experiments. However, the predicted docking pose for **1** in the D3R was not corroborated by the point mutation studies. This result suggested that **1** may adopt an alternative binding mode, and was further interrogated using unbiased, all-atom, large-scale molecular dynamics (MD) simulations. The use of MD to reconstruct ligand-receptor binding has been widely used in the last five years^2,17,18^, and proved to be valuable for characterizing binding modes while taking into account protein flexibility, e.g., in serine proteases^19,20^ and GPCRs^21^. Recently, it was successfully applied to reconstruct the protein-protein binding process of Barnase-Barstar^22^. The analysis performed in this work revealed 1) a different binding mode for compound **1** compared to poses generated by rigid docking, and 2) a cryptic pocket induced by compound **1** by displacement of the highly conserved residue F346^6.52^ between helices V and VI. Our study opens new opportunities for drug design in aminergic GPCRs and highlights the importance of protein dynamics in ligand binding.

## Results

### Mutagenesis and computational docking studies

**Figure 1:**
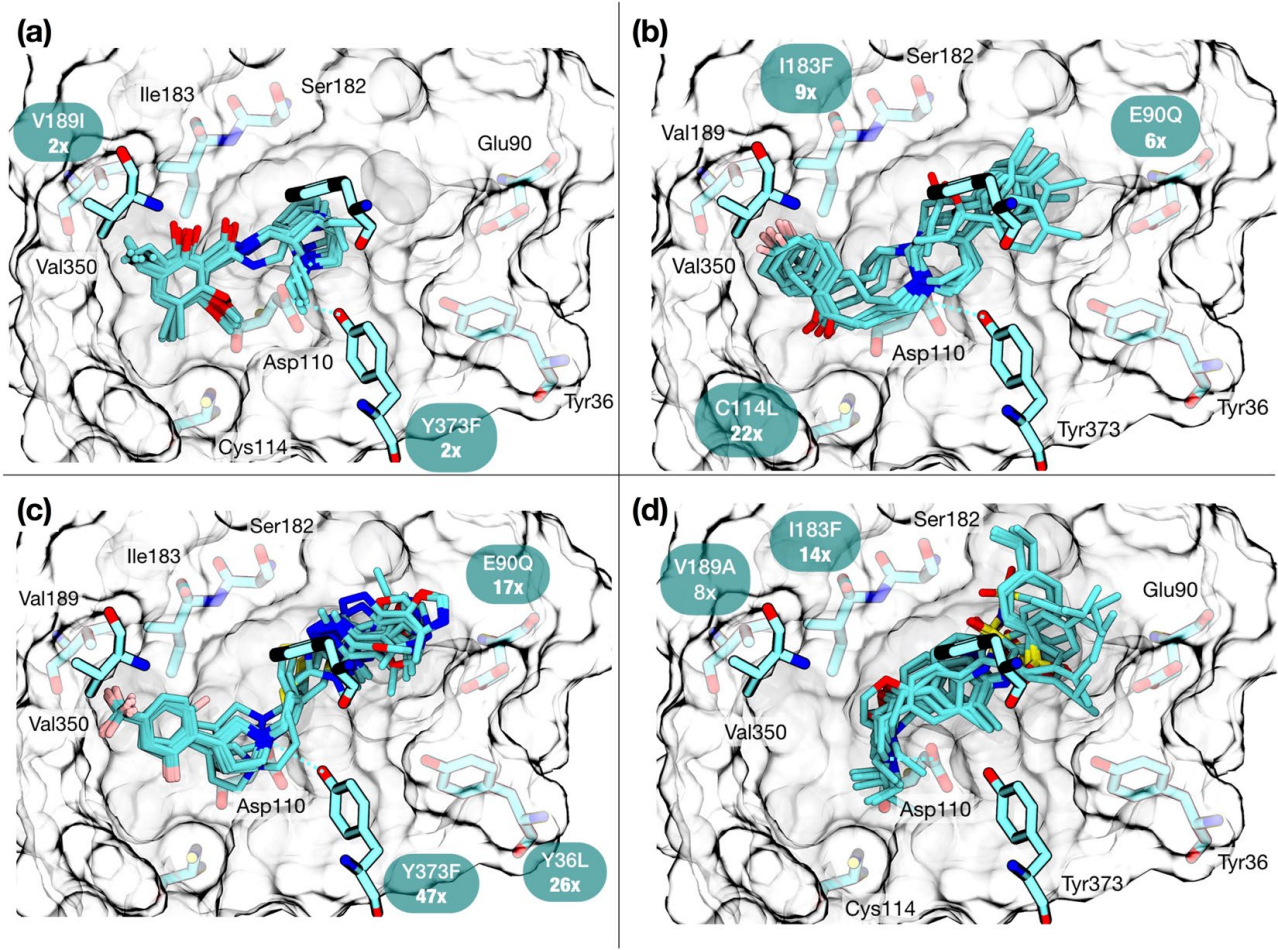
Binding modes for compounds 1–4 obtained from rigid docking using 3PBL as input coordinates for the receptor. The four structures highlight the same amino acids for direct comparison and the ten highest-scored docked poses. (**a**) 2 reproduces the crystal binding mode. Compounds 3 (**b**) and 4 (**c**) entirely occupy the OBS and extend towards the extracellular vestibule. (**d**) Compound 1 extends towards the extracellular vestibule.

The affinities of compounds **1–4** for 12 different D3R mutants were measured using radioligand binding experiments (Table S1). The D3R mutations were located in the OBS or the vicinity of the OBS and were performed on nine different residues (Fig. 1). The contribution of each residue to the binding affinities of **1–4** was determined by measuring their inhibition constants for the *wt*-D3R and mutant D3R forms. The impact of a mutation was evaluated by computing fold-shifts (*K*_i_^mutant^/*K*_i_^wt^), where values greater than one are considered indicative of a detrimental effect on the compound’s affinity.

To interpret the mutation data, compounds **1–4** were docked into the published D3R co-crystal structure (PDB ID: 3PBL) in complex with eticlopride^16^. The docking grid was created based on the eticlopride binding mode and enclosed the entire orthosteric pocket (see Methods). *A priori*, the binding mode of the compounds could be inferred from their similarities with eticlopride, as they all share interactions in the OBS. Three highly conserved^8^ ligand-aminergic receptor interactions were observed: 1) residue D110^3.32^ forms a salt bridge with the positively charged amino group of the ligands, 2) an aromatic cluster in helix VI W[Y]^6.48^ - F[Y]^6.51^ - F^6.52^ provides a subpocket for aromatic moieties, and 3) residues S^5.42^ and S^5.46^ interact with the hydroxyls of catecholamine agonists^23^. Fig. 1 summarizes the effect of these mutations superimposed with the docking predictions. For the full mutagenesis data set see Table S1 and Fig. S1.

Eticlopride (**2**), correctly re-docks into the X-ray structure (Fig. 1a). Its tertiary amine in the ethyl-pyrrolidine forms a salt-bridge with D110^3.32^, while the rest of the structure, kept almost planar due to intramolecular H-bonds, fits tightly in the hydrophobic pocket among helices III, V and the aromatic cluster in helix VI. Two mutations had minor effects on eticlopride affinity, namely, V189^5.39^I and Y373^7.43^F. The first mutation is located in the hydrophobic pocket, and the second directly interacts with the tertiary amine.

The binding mode of **3** has been the subject of intense research. Most studies agree on the region in which haloperidol binds in the OBS; however, there is not consensus on the orientation of the molecule, which diverges by 180°^24^. The most recent evidence from quantum mechanics^25^, molecular dynamics^24^ and experimental studies point to a binding mode with the butyrophenone moiety binding deep in the binding cavity^26^, in agreement with our docking prediction. The experiments suggest that the affinity of **3** is mainly affected by mutations C114^3.36^L, I183^ECL2^F and E90^2.65^Q (Fig. 1b). The effect of C114^3.36^ mutations on other D3R ligands has been tested in the past. In particular, the affinity of aminotetralin antagonists decreased significantly in the C114^3.36^S mutant form^27^. Reported QM-MM docking pose refinements on **3** also revealed the pivotal role of C114^3.36^ in binding^24^.

The predicted binding mode of **4** aligns with what has been previously reported^28^. The basic nitrogen of **4** forms a salt bridge with D110^3.32^ while the substituted phenyl ring fills the hydrophobic region in the OBS. In addition, the terminal oxazole ring of the aromatic group clearly points toward the SBP in the extracellular opening. Mutations that most affected the binding of compound **4** were Y36^1.39^L, E90^2.65^Q, and Y373^7.43^F. These residues are located at the cavity between helices I, II and VII where the biaryl system binds (Fig. 1c). Whereas Y36^1.39^ and Y373^7.43^ do not form specific interactions with **4** and may share a structural role in D3R, the loss of binding affinity observed with the E90^2.65^Q mutation could be explained by the disruption of the favorable electrostatic interaction between the acidic CH in the oxazole moiety and the carboxyl group of E90^2.65^.

The docked pose for **1** suggests a salt bridge between D110^3.32^ and the positively charged nitrogen in the morpholine ring (Fig. 1d). The 4-isopropylphenyl group occupies a crevice enclosed by the extracellular loops (also termed the extracellular vestibule), interacting with a cluster of hydrophobic and aromatic residues: L89^2.64^, V95^ECL1^, F106^3.28^, C181^ECL2^, and Y365^7.35^. Remarkably, this binding mode leaves unoccupied most of the hydrophobic region in the OBS delimited by helix III, V and VI. Mutation of I183^ECL2^ into Phe resulted in a 14-fold decrease in affinity. A second detrimental mutation located in the same subpocket, V189^5.39^A, was accompanied by an 8-fold decrease in affinity. These changes in binding affinity could be explained if compound **1** filled the region adjacent to I183^ECL2^ - V189^5.39^: the introduction of a larger residue with the I183^ECL2^F mutation would clash with the compound, and reducing the sidechain size with the V189^5.39^A mutation would potentially lose favorable hydrophobic interactions. However, in the binding mode predicted by docking, compound **1** is only partially filling this region and positioned more than 6 Å and 9 Å away from the I183^ECL2^F and V189^5.39^A mutations, respectively. While most of the mutations seem to agree with our predicted binding mode, these two are exceptions and stand out as incompatible with the docking model for **1**.

### Molecular dynamics simulations and Markov state models of PF-4363467/D3R complex

To further interrogate the binding mode of **1** with D3R, high-throughput unbiased MD simulations were performed. This approach had initially been validated using **2**, for which the binding mode at D3R is known, as a control experiment (details in **Text S1** and Fig. S2). In total, 700 μs of aggregated simulation time were produced, and **1** was observed to spontaneously bind to D3R in multiple different poses in this timeframe. The simulations were run on the distributed computing project GPUGRID.net^29^ using ACEMD^30^ directed sampling and the Markov state model (MSM) analysis with HTMD^31^. We used an unsupervised adaptive sampling protocol^32^ that allows for efficient exploration of conformational space without biasing the dynamics of the system by performing simulations in successive epochs. At each epoch, the cumulative ensemble of simulations is automatically analyzed using MSMs from which the starting conformations for the following epoch of simulations are identified (see Methods).

Initially, a set of exploratory simulations composed of short trajectories of 25 ns was run, accumulating an aggregate of 259 μs. During the simulations, the ligand freely diffused from bulk and initiated interactions at different locations in the receptor. Over the course of the successive simulation epochs, the MSM analysis provided intermediate metastable binding modes (Fig. S3). Subsequently, a second set of 100 ns trajectories was run. In this case, the ligand was located in bulk in 50% of the starting points and was in contact with the protein following the previous analysis’ equilibrium distribution in the other 50% (Text S2).

**Figure 2:**
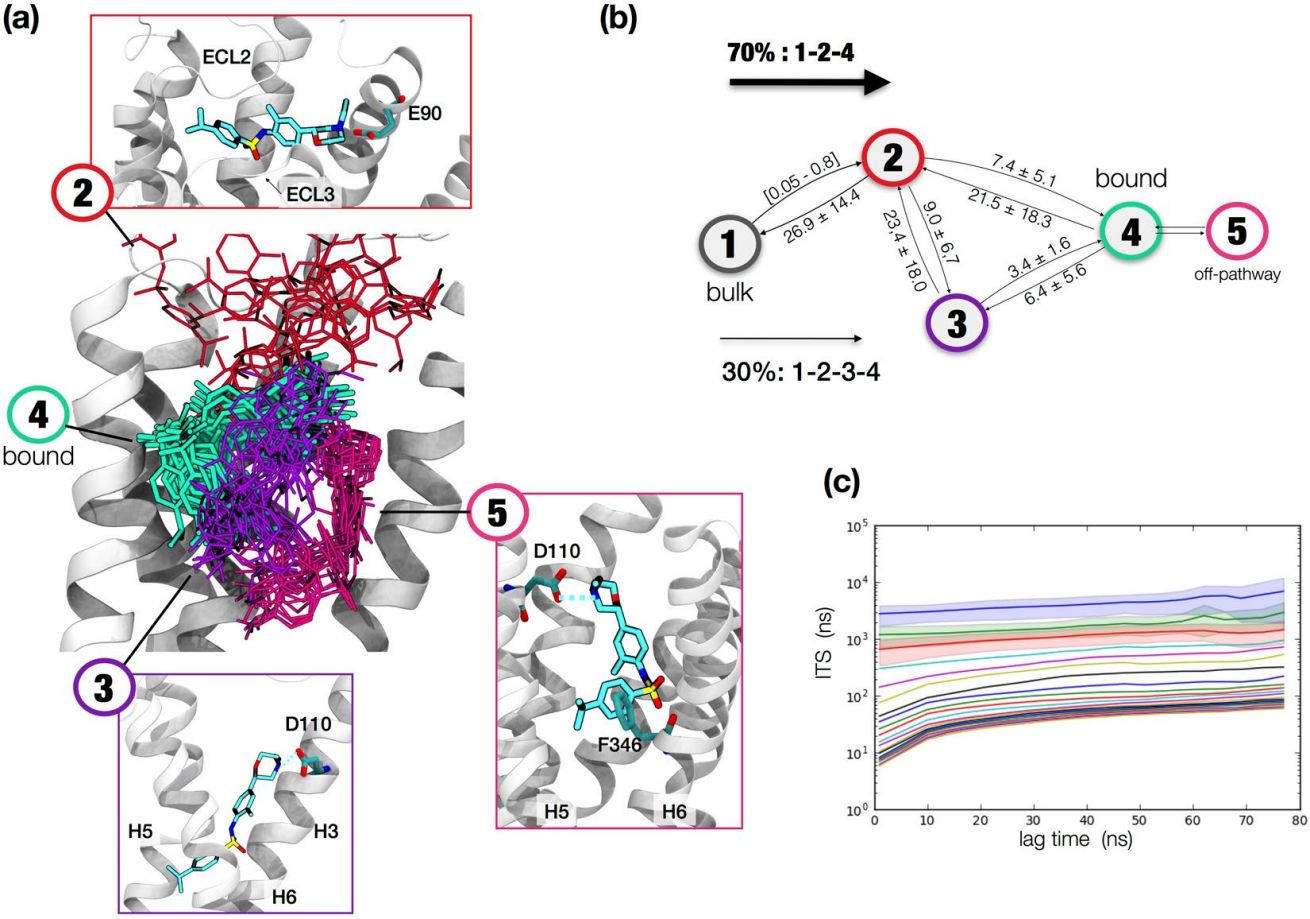
Details of the MSM analysis. (**a**) The MSM produced 5 states. State 1, bulk, is not shown for clarity. State 2 binds to the extracellular vestibule while poses 3–5 occupy the OBS. Bound state is shown in Fig. 3 in detail. (**b**) Transitions between states before reaching bound, in microseconds. (**c**) The implied timescales of the MSM.

In total, 220 μs from the second set of trajectories were used for this analysis. The data was separated into 800 clusters that were then kinetically lumped together into five states^33^. These states corresponded to the basins of the complex energy landscape, i.e. to bulk, intermediate and bound poses in the ligand binding pathway (Fig. 2a). State 1 corresponds to bulk, or the initial state in the reaction pathway, when the ligand is free in solution. State 2 is located at the extracellular vestibule and corresponds to the first state when the ligand initiates contacts with the protein. It recognizes E90^2.65^, in agreement with a recent study on the binding pathways of clozapine and haloperidol to D2R and D3R^24^. State 3 is a broad state in the OBS that encompasses several meta-stable poses before reordering in the final bound pose, state 4. Finally, state 5 is an off-pathway state that does not convert to other states without reverting to bound.

The whole process of binding occurred with a mean first passage time (MFPT_on_) of 7.0 ± 5.0 μs, which corresponded to a k_on_ of (12 ± 8) 10^6^ M^−1^ s^−1^, in line with previous works on drug binding to GPCRs^2^. Compound **1** can reach the bound state through two different interconnected pathways of binding (Fig. 2b). In the fastest and most common route of binding, compound **1** must traverse two energetic barriers. The first step consists of a diffusion-limited process where **1** recognizes the extracellular vestibule in the nanosecond timescale, ranging from around 50 to 800 ns. The second, rate-limiting step occurs in 7.4 ± 5.1 μs and comprises the recognition between the charged amine in **1** and D110^3.32^, followed by a rearrangement of the complex to reach the bound state (Fig. 2b). The second, less populated pathway occurs in 30% of the binding processes. It shares the first step with the previous pathway, but includes an additional intermediate state of binding between the extracellular pose and the bound state. Conversion between extracellular state 2 and the alternate bound state 3 occurs in an average time of 9.0 ± 6.7 µs, which is then followed by a reordering in the pocket toward the bound state, crossing a lower energetic barrier (3.4 ± 1.6 µs). An example of a binding trajectory is shown in Movie S1. The three states in complex with the receptor present delta free energies of binding of the order of 4.8–5.4 kcal/mol. The MSM is therefore underestimating the binding free energy (ΔG_exp_ = −11.6 kcal/mol). State 4 is the most probable, and although states 2 and 3 have free energies comparable within the error bars, state 4 provides better alignment with the mutation data.

**Figure 3:**
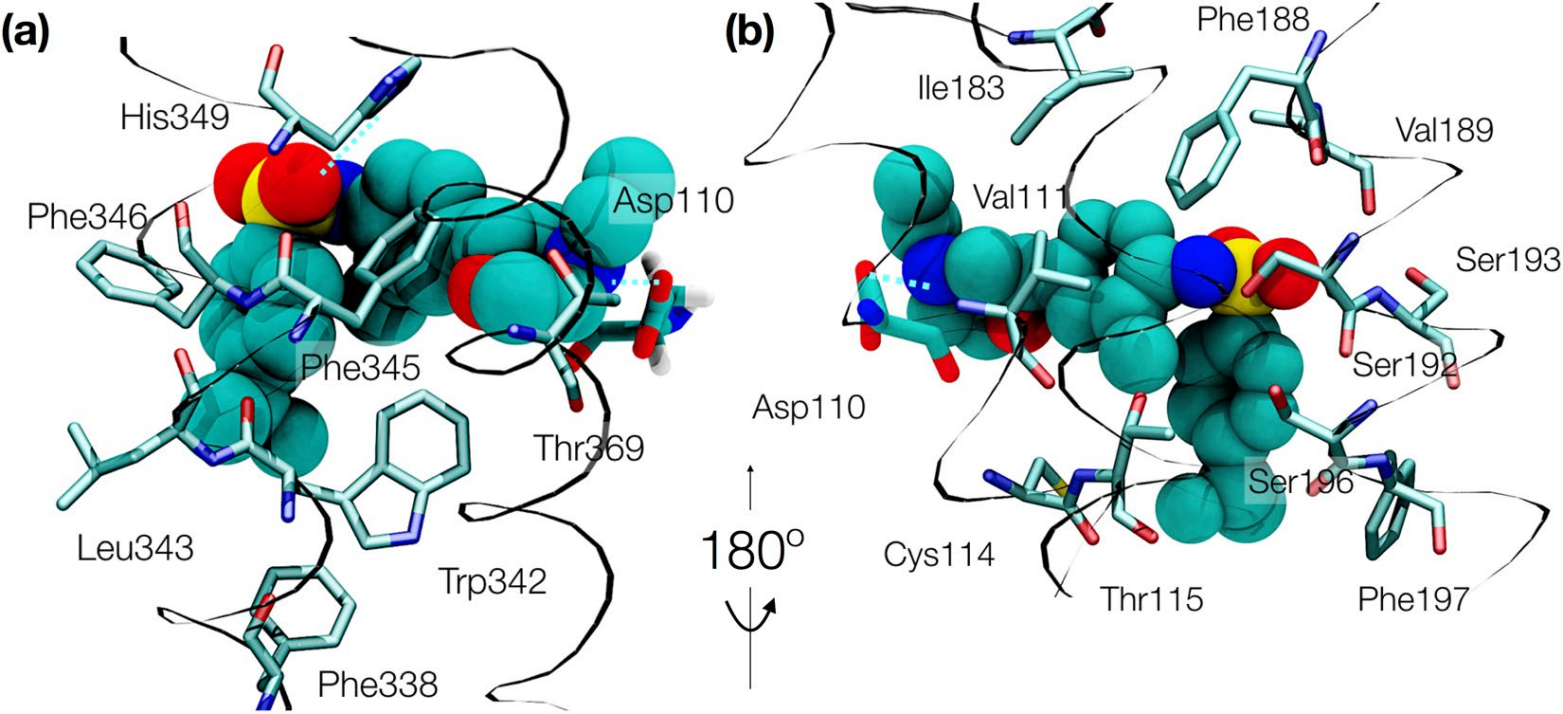
Binding mode of compound 1. Frontal (**a**) and back (**b**) views of the ligand pose and interacting residues within 4 Å. Polar interactions are depicted by blue dashed lines. Asp110 is shown in both representations as reference.

**Figure 4:**
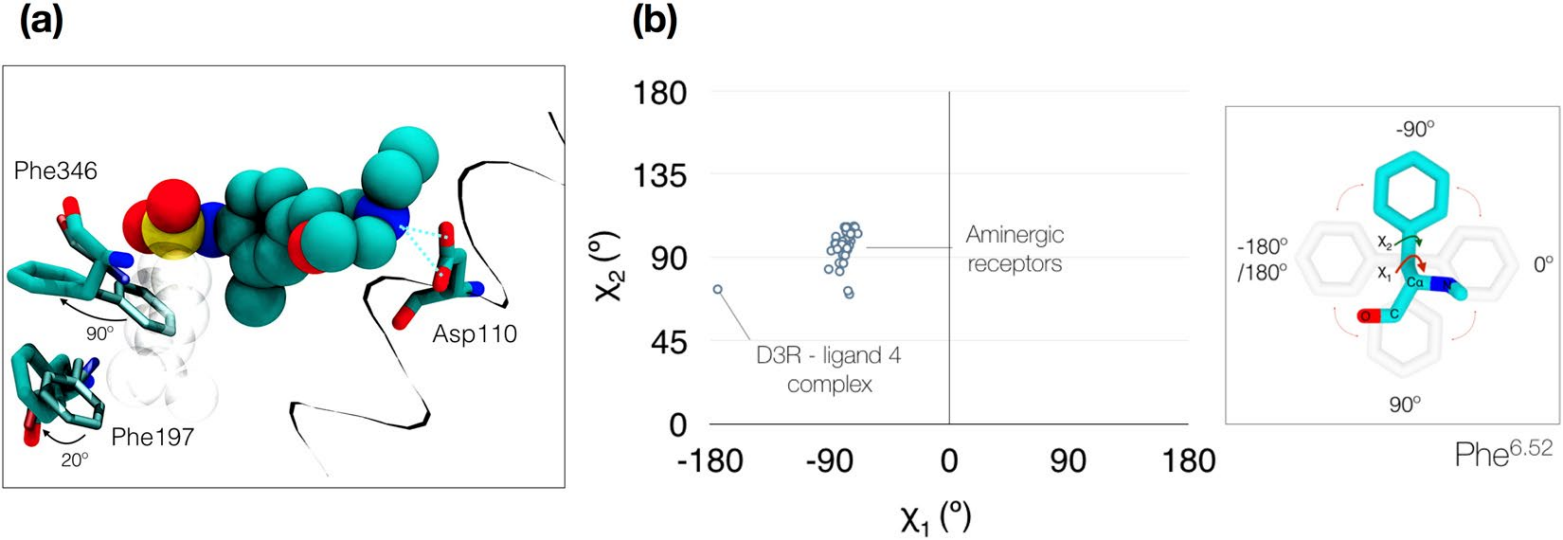
Opening of a cryptic-pocket after displacement of F197^5.47^and F346^6.52^ by compound 1. (**a**) Displacement of the two phenylalanine residues in the D3R:**1** complex versus the X-ray crystal structure by the 4-isopropylphenyl group in **1** (shown in transparent grey). (**b**) Structural analysis performed for the phenylalanine sidechain dihedrals (χ_1_ and χ_2_) as represented in the inset diagram. The conformation stabilized by compound **1** explores a χ_1_ angle close to −180°, previously unseen in the studied set of 39 crystallized aminergic receptors, which are close to −90°.

From the almost 12,000 frames belonging to state 4, the pose with the closest RMSD to the pocket side chains from the available X-ray structure (3PBL) was selected for visualization. The interactions show that all of the heavy atoms of **1** are in contact with 19 residues at a distance of 4 Å or less (Fig. 3). The primary amine in the morpholine ring forms the conserved salt bridge with D110^3.32^ while the rest of the ring is packed between residues T369^7.39^ and V111^3.33^. The 2-methylphenyl group packs between residues V111^3.33^, C114^3.36^, I183^ECL2^ and F345^6.51^ with the methyl substituent oriented toward T115^3.37^. The sulfonamide oxygen interacts with conserved S192^5.42^, S193^5.43^ and H349^6.55^, which together with I183^ECL2^ and Y365^7.35^, define the surface curvature of the upper side of the D3R ligand binding pocket. In contrast to the D3R X-ray crystal structure with **2**, the 4-isopropylphenyl group displaces the aromatic residues F346^6.52^ and F197^5.47^ (Fig. 4a), opening a cryptic pocket delimited by residues F338^6.44^, W342^6.48^, L343^6.49^, F345^6.51^, and F346^6.52^.

The predicted protein conformation of D3R in complex with **1** does not significantly differ from the experimentally determined co-crystal structure of D3R with **2**, or with the recently crystallized D4R structure with the antagonist Nemonapride:^34^ overlays of the Cα backbone atoms with these two crystal structures result in RMSDs of 2.1 and 3.3 Å, respectively. The ionic lock salt bridge between R^3.50^ in the conserved D[E]RY motif and D/E^6.30^, a common feature in many GPCR structures and conserved in rhodopsin and D3R/D4R inactive structures, is also present in the predicted D3R structure with **1**. The distance between the E^6.30^ oxygen and R^3.50^ nitrogen atoms is 2.7 Å, in line with the D3R and D4R X-ray structures (2.5 Å and 4.5 Å respectively). ICL2 is helical in the predicted structure of D3R with **1**, similar to that seen in chain A of the D3R X-ray crystal structure with **2**. The conserved NPxxY motif, recognized as a critical determinant of GPCR activation^35^ superimposes with both D3R and D4R structures. Y^7.53^ in the NPxxY motif plays a key role in GPCR activation, which takes part in the so-called tyrosine toggle switch along with Y^5.58 ^^36^. During activation, the two sidechains rearrange and disrupt the ionic lock, whereas in the antagonist-induced structures of D3R and D4R, they are 10.7 Å and 9.7 Å apart, respectively. We observe that Y^7.53^ occupies a different rotamer state in our predicted D3R:**1** structure, leading to a distance of 18.3 Å, in line with the recent MD study of D3R:**3** complex^36^.

Aside from the different rotamer states in the tyrosine toggle switch, the formation of the aromatic cryptic pocket between helices V and VI comprises the greatest conformational difference between the predicted D3R:**1** structure and the D3R:**2** X-ray crystal structure. Residues F197^5.47^ and F346^6.52^ are pushed away from their original X-ray positions by 20 and 90 degrees, respectively, allowing the 4-isopropylphenyl group to fit deeper in the OBS. To our knowledge, such a conformation has not been observed in published crystal structures of other aminergic GPCRs. In order to validate this assumption, we performed a structural superimposition of publicly available aminergic receptors. From the six existing aminergic subfamilies, four of them present a phenylalanine at position 6.52, totaling six representative receptors with X-ray data: serotonin (5HT_1B_, 5HT_2B_), adrenergic (β1 A, β2 A), dopamine (D3R), and histamine (H1R). We used 39 available crystal structures for our analysis (Table S2). After superimposing all crystal structures, we measured the phenylalanine sidechain dihedral angles χ_1_ and χ_2_, which provided distributions of (−90.8°, −68.9°) and (70.2°, 106.8°), respectively. The value for χ_2_ in our binding mode is within this array (72.9°), but the χ_1_ value lies outside of the previously observed conformations (−174.4°) (Fig. 4b). While this new conformation has not been detected in any solved X-ray co-crystal structure to date, we found a similar conformation of this aromatic pocket in a recently disclosed large-scale MD study by Thomas *et al*.^24^. Although the authors did not discuss this explicitly in the manuscript, we were able to find a pose of clozapine bound to D3R within the Supplementary Information that is consistent with the F346^6.52^ rotamer identified in this study.

**Figure 5:**
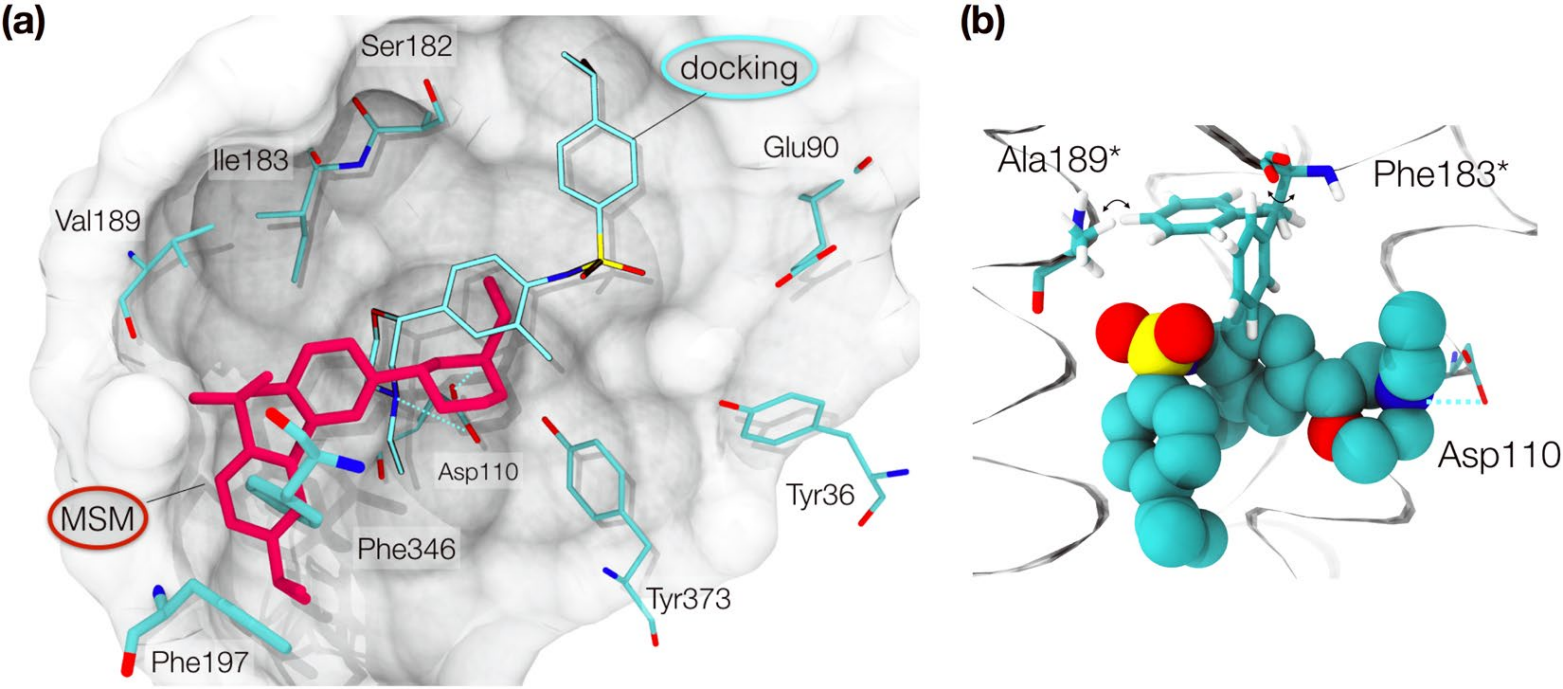
Comparison of docking and MSM poses and effect of mutations. (**a**) Overlay of the pose obtained by high-throughput molecular dynamics (red), the docking prediction (blue) and the residues implicated in binding in the 3PBL coordinates. The new pose obtained by MSM fills the pocket formed by V189^5.39^A, and I183^ECL2^F and would clash with F197^5.47^and F346^6.52^ in their crystal structure coordinates. (**b**) The pose obtained by molecular dynamics provides a rationale for the effect of the two mutations (V189^5.39^A, I183^ECL2^F). The mutated residues are shown with an asterisk. Rotation of the engineered F183^ECL2^ would clash with either the ligand or residue 189, affecting protein stability and ligand affinity. Mutant A189^5.39^ would lose favorable packing (van der Waals) interactions by reduction of its sidechain.

The pose identified for **1** based on this extensive simulation data provides a rationale for the potency loss observed with the I183^ECL2^F and V189^5.39^A mutations in the OBS. The superposition of the poses obtained by docking and the simulation data along with the performed mutations is shown in Fig. 5a. Although both poses interact with D110^3.32^ via the morpholine salt bridge, they adopt very different orientations. The docking pose (blue) extends towards the extracellular vestibule, while the simulation data pose (red) fits tightly into the OBS and opens a cryptic-pocket among helices V and VI. The sidechain of V189^5.39^ is in contact with the sulfonamide group in **1**, and the heavy-atom reduction to Ala decreases favorable hydrophobic interactions. Mutation of a small, neutral amino acid such as I183^ECL2^ to a larger one such as Phe would have detrimental effects by clashing with compound **1** as shown in Fig. 5b. Indeed a phenylalanine at this position would either clash with the 2-methylphenyl group or with the nearby sidechain of V189^5.39^.

## Discussion

In this work, the binding poses for compounds **1–4**, which are known to have affinity for D3R, were studied. Docking poses generated for compound **1** were not consistent with results from mutagenesis experiments, suggesting it may adopt a different pose compared to **2** in the D3R X-ray co-crystal structure. By performing high-throughput molecular dynamics simulations, a new binding mode was identified that was consistent with mutation results. This pose stabilized an inactive D3R conformation by displacing F346^6.52^ to a conformation that has not yet been observed in aminergic GPCRs. Compound **1** binds by opening a cryptic pocket, which can be exploited in the design of D3R antagonists and, by extension, other aminergic GPCR ligands.

The recent advances in GPCR structural biology have led to unprecedented high-resolution pictures of GPCR-ligand complexes. These structures have been of tremendous importance in drug design^34^, with some of these efforts leading to drugs currently in clinical trials. This work suggests that characterizing the intrinsic flexibility of GPCRs is of considerable importance in structure-based drug design (SBDD), even for sampling different inactive conformational states. Docking of compound **1** or analogous compounds into the previously crystallized D3R structure would have missed this promising compound in a virtual screening protocol, as single-structure GPCR SBDD only provides conformational accessibility to a subset of ligands^37^. In the near future, we expect that approaches similar to the one presented here will become common in the preclinical stage of the drug discovery pipeline and will be particularly enabling for GPCR preclinical programs where obtaining X-ray structures is challenging.

## Methods

### D3R construct

The wild type template D3R construct was a kind gift from Ellen Y. T. Chien^16^, subcloned into a pFastbac vector containing a polyhedran promoter. Full sequence is presented in Text S3.

### Mutagenesis

Point mutants were made by site-directed mutagenesis using 0.5 μl Takara’s PrimeStar Hot start polymerase with 20ng D3R wild type template plasmid DNA, 5 × PrimeStar buffer, 4 μl dNTP mix (2.5 mM each dNTP), plus 0.5 μg forward and reverse primers. 18 cycles were performed at 98 °C for 30 seconds, 55 °C for 30 seconds and 72 °C for 6.5 minutes. The template was degraded with DpnI for 1 hr and transformed into DH5α competent cells.

### Expression

Mutants were expressed in SF9 insect cells for 48 hrs with an approximate MOI of 5. A list of mutagenesis primers used can be found in Table S3.

### ***In Vitro*** Receptor Binding Assay

Binding assays were performed on membranes prepared from transiently transfected human D3 receptor mutants in SF9 insect cells. Frozen cell paste was homogenized by Polytron in 50 mM Tris HCl buffer (pH 7.4 at 4 degrees C) containing 2.0 mM MgCl_2_. The cell membranes were spun in a high-speed centrifuge at 40,000 × g for ten minutes. The final pellet was re-suspended in assay buffer (50 mM Tris buffer, 120 mM NaCl, 5 mM MgCl2, 5 mM KCl, 2 mM CaCl2, pH 7.4 at 37 °C). Incubation was initiated by the addition of cell membrane solution to 96-well plates containing test compounds and [^3^H] 7-OH-DPAT (1 nM final concentration). The final concentration of protein in the assay was between 2 and 30 μg/well, depending on receptor expression of the transient mutant. Non-specific binding was determined by the presence of a saturating concentration (10 μM) of **3**, a potent D3 antagonist. After a 20 minutes incubation period at 37 °C, assay samples were rapidly filtered through PEI coated, GF/B fired Unifilter plates (PerkinElmer) and rinsed with ice-cold 50 mM Tris buffer (pH 7.4). Filterplates were allowed to dry overnight. Membrane bound [^3^H] 7-OH-DPAT levels were determined by liquid scintillation counting of the filterplates in Ecolume scintillation fluid. The IC_50_ value (concentration at which 50% inhibition of specific binding occurs) was calculated by curve fitting of the concentration response, percent inhibition data. K_i_ values were calculated according to the Cheng- Prusoff equation, K_i_ = IC_50_/(1 + (L/K_d_)), where L is the concentration of the radioligand used in the experiment and the K_d_ value is the dissociation constant for the radioligand at the mutant receptor (determined previously by saturation analysis).

### Docking

Molecular docking experiments were performed with GLIDE (version 6.9 Schrödinger package)^38–40^. The protein 3D model was prepared using the Schrödinger Protein Preparation Wizard workflow^41^, starting from the D3R X-ray structure 3PBL and adding hydrogens at pH = 7.0. Taking the prepared receptor, Glide docking experiments were performed following receptor grid generation. The center of the grid box was defined by the center of the bound ligand as described in the original PDB entry. The enclosing box dimensions, automatically deduced from the ligand size, fit the entire active site. Concrete dimensions were 10 × 10 × 10 and 40 × 40 × 40 Å, for the inner and outer boxes, respectively. The standard precision mode (SP) of Glide was chosen, sampling ligands as flexible, saving 10 maximum structures per ligand and setting D110 as H-bond donor constraint. A post-docking optimization of the docking poses was performed choosing 0.5 kcal/mol as the cutoff for rejecting the minimized poses. For each processed compound, we considered the best scoring docking model for comparison with the mutagenesis and MD data. Flexible docking was also performed using Schrödinger´s Induced Fit Docking (IFD) Workflow^42,43^. IFD initial docking was done by Glide set to standard precision. The docking site was also defined by the original ligand and the receptor optimization by Prime was set to the residues within 5 Å from **2**, excluding those forming hydrogen bonds with the ligand.

### System setup and MD production

Systems were built, equilibrated and run with the software HTMD^31^. The protein coordinates were taken from the PDB code 3PBL^16^. L119 was mutated to Trp according to the wild type sequence. The lysozyme T4 protein was removed for the simulation and the resulting gap in the third cytoplasmic loop (ICL3) capped, by addition of neutral caps at residues 220 and 320. Protonation states were determined with the propKa tool at pH = 7.2^44^. The membrane was formed by a 80 × 80 A^2^ POPC lipid bilayer and its location was set using the OPM database^45^. The ligand was randomly placed around the protein in the extracellular part, with random x and y coordinates while keeping the z coordinate at 43 Å. The system was parameterized with the Charmm36 forcefield for the protein atoms and lipids^46^. Parameterization of the ligand was performed using a derivative of the Gaamp tool^47^ implemented in HTMD (Fig. S4). The system was solvated using TIP3P water molecules^48^ and neutralized with NaCl at a 0.150 M concentration, giving a total of 48650 atoms. Four different systems were built and used as input for four independent minimizations and equilibrations.

Energy minimization and equilibration were conducted under NPT conditions at 1 atm, 298 K, using a time step of 4 fs for thermalization and a cutoff of 9 Å, with rigid bonds and PME for long-range electrostatics. Energy minimization was run for 250 fs and equilibration for volume relaxation was run for 40 ns. In these two phases, the heavy protein atoms were restrained by a 1 kcal·mol^−1^·Å^−2^ spring constant that linearly decreased until 20 ns, when it was set to zero. During the production runs, a flat-bottom potential was set using a cuboid box with dimensions [−20, 20, −20, 20, −20, 35] for the x1, x2, y1, y2, z1, and z2 coordinates, respectively. Whenever the ligand explored an area outside the box, it was pushed back with a force proportional to its distance from V111^3.33^’s alpha carbon and a constant of 5 kcal/mol·Å^2^. The flat-bottom potential kept the ligand from crossing to other periodic boxes, and from inefficiently exploring the receptor’s intracellular region or the lipid bilayer for large times. The effective concentration of the simulation box computes as 18.9 mM, taking into account the volume of the flat-bottom box (88 nm^3^). Minimizations and equilibrations were performed on local workstations whereas the productions were run with ACEMD^30^ on GPUGRID.net^29^. By using an adaptive sampling protocol^32^, a total of 680 μs were run from which 220 μs were used for the analysis in the main text (Text S2).

Simulation data and binding pose may be distributed upon request.

### Markov State Models

Markov modeling proceeds from the discretization of the conformational space of the system of interest and the description of the dynamics of the system as a sequence of transitions between all discrete states. An optimally discretized MSM shows converging timescales with high probability of transition among kinetically similar states, and lower probability between kinetically separated states. From this model, the pathways and kinetic rates between distinct conformations may be derived^49^. In this case the conformations were represented by means of two separate protein-ligand contact representations. The first is a contact map, which accounts for interactions of all residues in the protein with the ligand. The distances between all heavy atoms of the protein to all oxygens, nitrogens and carbons not attached to hydrogens in the ligand are calculated. If any residue has a distance of less than 5 Å from the considered ligand atoms, the residue is considered to be in contact with the ligand. The second contact representation is a single flag, which is set to true if any of the above-mentioned atoms in the ligand is closer than 10 Å to any CA atom in the protein, thus creating an extra dimension which can be used to separate the bulk from the interacting states.

One of the most important requirements for constructing Markov models is to be able to finely discretize the slowest order parameters. TICA^50^ (time-structure independent component analysis) is a method that allows projecting the data on the slow order parameters, yielding very good discretization. After projecting the first protein-ligand contact representation onto the five slowest processes found by TICA, the 5-dimensional projected data was clustered into 800 clusters using the mini batch k-means algorithm^51^. Although this fine discretization provides very accurate Markov models, it is necessary to reduce the amount of states to obtain a humanly interpretable model of the system in question. Hence, the produced clusters were then lumped together into 4 macrostates using the PCCA algorithm^33^, each consisting of a set of kinetically similar clusters. Next, the protein-ligand contact-flag used to indicate bulk states was used to split a 5^th^ bulk state out of the other 4 macrostates to obtain cleaner kinetics of binding. Finally, core sets were used to improve the model quality. This was done by identifying clusters that belonged to the cores of the 5 macrostates and recalculating the Markov model accounting only for transitions between the core clusters. This provided improved estimates of kinetics between states, as spurious transitions between states caused by inaccuracies in cluster borders are discounted. The converged timescales of the final model can be seen in Fig. 2c.

## Electronic supplementary material

Supplementary Information

Supplementary Movie S1
