# Author Correction: Dopamine D3 receptor antagonist reveals a cryptic pocket in aminergic GPCRs

**DOI:** 10.1038/s41598-019-39694-1

**Published:** 2019-04-10

**Authors:** Noelia Ferruz, Stefan Doerr, Michelle A. Vanase-Frawley, Yaozhong Zou, Xiaomin Chen, Eric S. Marr, Robin T. Nelson, Bethany L. Kormos, Travis T. Wager, Xinjun Hou, Anabella Villalobos, Simone Sciabola, Gianni De Fabritiis

**Affiliations:** 1 Computational Biophysics Laboratory (GRIB-IMIM), Universitat Pompeu Fabra, Barcelona Biomedical Research Park (PRBB), Doctor Aiguader 88, 08003 Barcelona, Spain; 2 Acellera, PRBB, Doctor Aiguader 88, 08003 Barcelona, Spain; 3 Institució Catalana de Recerca i Estudis Avançats (ICREA), Passeig Lluis Companys 23, 08010 Barcelona, Spain; 4 Pfizer Worldwide Research and Development, 1 Portland Street, Cambridge, Massachusetts, 02139 United States; 5 Pfizer Worldwide Research and Development, Eastern Point Road, Groton, Connecticut 06340 United States

Correction to: *Scientific Reports* 10.1038/s41598-018-19345-7, published online 17 January 2018

We realized that there is a mistake in the presentation of the ligands which might affect the understanding of the paper. Compound **1** in Table 1 exists as two separate enantiomers R (PF-4363476) and S (PF-4363467), as reported in a previous paper published by us^15^. In the current paper we used a single name (‘compound **1’**) to refer to both stereoisomers, while we should have specified which form was used exactly when. Both forms have similar activity experimentally (Table 1) and identical docking poses (Fig. 1) but they are chemically different. The updated Table 1 reflects this difference and specifies that **1S** was used in the mutagenesis experiments while **1R** was used for both rigid docking and simulation work. For simplicity, only the 2D depiction of the R-enantiomer of **1** is shown in Table 1. We now use ‘Compound **1’** to refer to both forms while we indicate 1R and 1S when this is relevant.

**Table 1:** Summary of compounds studied in this work.

ID	Name	2D structure	*K*_i_ (nM)	Mutations
1*	PF-4363467 **(1S)** PF-4363476 **(1R)**		3.4 ± 0.4 2.4 ± 0.6	I183^ECL2^F V189^5.39^A
**2**	Eticlopride		0.24^16^	V189^5.39^I Y373^7.43^F
**3**	Haloperidol		6.5 ± 1.0	C114^3.36^L, I183^ECL2^F E90^2.65^Q
**4**	GSK598809		2.5 ± 0.4	Y36^1.39^L E90^2.65^Q Y373^7.43^F

Structure, inhibition constant (*K*_i_), and mutations that most affected binding for each compound. Values presented were measured in this work except eticlopride (**2**), measured in ref.^16^. *Compound 1 exists as two enantiomers; **1S** was used in the mutagenesis experiments and **1R** was used for both rigid docking and simulation work. For simplicity, only the 2D depiction of the **R**-enantiomer of **1** is shown. In the manuscript we use compound **1** to refer to both forms while we indicate **1R** and **1S** when this is relevant.

**Figure 1:**
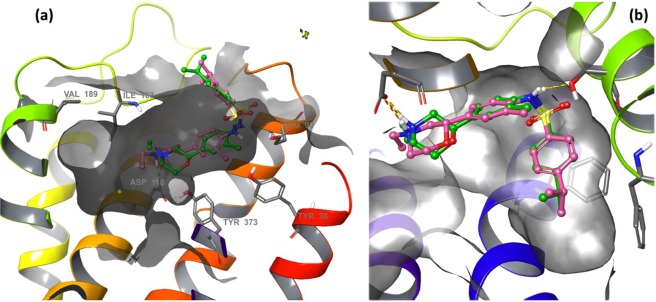
Proposed binding mode of **1R** and **1S** to D3R. (**a**) Predicted binding mode for **1R** and **1S** obtained using rigid docking and the 3PBL structure as input coordinates for the receptor. (**b**) The **D3R:1R** receptor conformation identified in state 4 of the MSM model was used to rigidly dock compound **1S** (pink) producing an identical docking pose. Both enantiomers have similar activity at D3R experimentally (Table 1) and share equivalent docking poses.

As a result, in the Introduction,

“However, the predicted docking pose for **1** in the D3R was not corroborated by the point mutation studies.”

should read:

“However, the predicted docking pose for **1R** in the D3R was not corroborated by the point mutation studies.”

In the Results section,

“The docked pose for **1** suggests a salt bridge between D110^3.32^ and the positively charged nitrogen in the morpholine ring (Fig. 1d).”

should read:

“The docked pose for **1R** suggests a salt bridge between D110^3.32^ and the positively charged nitrogen in the morpholine ring (Fig. 1d).”

“Molecular dynamics simulations and Markov state models of PF-4363467/D3R complex”

should read:

“Molecular dynamics simulations and Markov state models of 1R/D3R complex”

“To further interrogate the binding mode of **1** with D3R, high-throughput unbiased MD simulations were performed.”

should read:

“To further interrogate the binding mode of **1R** with D3R, high-throughput unbiased MD simulations were performed.”

“In total, 700 μs of aggregated simulation time were produced, and **1** was observed to spontaneously bind to D3R in multiple different poses in this timeframe.”

should read:

“In total, 700 μs of aggregated simulation time were produced, and **1R** was observed to spontaneously bind to D3R in multiple different poses in this timeframe.”

“Compound **1** can reach the bound state through two different interconnected pathways of binding (Fig. 2b). In the fastest and most common route of binding, compound **1** must traverse two energetic barriers. The first step consists of a diffusion-limited process where **1** recognizes the extracellular vestibule in the nanosecond timescale, ranging from around 50 to 800 ns. The second, rate-limiting step occurs in 7.4 ± 5.1 μs and comprises the recognition between the charged amine in **1** and D110^3.32^, followed by a rearrangement of the complex to reach the bound state (Fig. 2b).”

should read:

“Ligand **1R** can reach the bound state through two different interconnected pathways of binding (Fig. 2b). In the fastest and most common route of binding, it must traverse two energetic barriers. The first step consists of a diffusion-limited process where it recognizes the extracellular vestibule in the nanosecond timescale, ranging from around 50 to 800 ns. The second, rate-limiting step occurs in 7.4 ± 5.1 μs and comprises the recognition between the charged amine in **1R** and D110^3.32^, followed by a rearrangement of the complex to reach the bound state (Fig. 2b).”

“The interactions show that all of the heavy atoms of **1** are in contact with 19 residues at a distance of 4 Å or less (Fig. 3).”

should read:

“The interactions show that all of the heavy atoms of **1R** are in contact with 19 residues at a distance of 4 Å or less (Fig. 3).”

“The predicted protein conformation of D3R in complex with **1** does not significantly differ from the experimentally determined co-crystal structure of D3R with **2**, or with the recently crystallized D4R structure with the antagonist Nemonapride^34^: overlays of the Cα backbone atoms with these two crystal structures result in RMSDs of 2.1 and 3.3 Å, respectively. The ionic lock salt bridge between R^3.50^ in the conserved D[E]RY motif and D/E^6.30^, a common feature in many GPCR structures and conserved in rhodopsin and D3R/D4R inactive structures, is also present in the predicted D3R structure with **1**. The distance between the E^6.30^ oxygen and R^3.50^ nitrogen atoms is 2.7 Å, in line with the D3R and D4R X-ray structures (2.5 Å and 4.5 Å respectively). ICL2 is helical in the predicted structure of D3R with **1**, similar to that seen in chain A of the D3R X-ray crystal structure with **2**.”

should read:

“The predicted protein conformation of D3R in complex with **1R** does not significantly differ from the experimentally determined co-crystal structure of D3R with **2**, or with the recently crystallized D4R structure with the antagonist Nemonapride:^34^ overlays of the Cα backbone atoms with these two crystal structures result in RMSDs of 2.1 and 3.3 Å, respectively. The ionic lock salt bridge between R^3.50^ in the conserved D[E]RY motif and D/E^6.30^, a common feature in many GPCR structures and conserved in rhodopsin and D3R/D4R inactive structures, is also present in the predicted D3R structure with **1R**. The distance between the E^6.30^ oxygen and R^3.50^ nitrogen atoms is 2.7 Å, in line with the D3R and D4R X-ray structures (2.5 Å and 4.5 Å respectively). ICL2 is helical in the predicted structure of D3R with **1R**, similar to that seen in chain A of the D3R X-ray crystal structure with **2**.”

“We observe that Y^7.53^ occupies a different rotamer state in our predicted D3R:**1** structure, leading to a distance of 18.3 Å, in line with the recent MD study of D3R:**3** complex^36^.”

should read:

“We observe that Y^7.53^ occupies a different rotamer state in our predicted D3R:**1R** structure, leading to a distance of 18.3 Å, in line with the recent MD study of D3R:**3** complex^36^.”

“Aside from the different rotamer states in the tyrosine toggle switch, the formation of the aromatic cryptic pocket between helices V and VI comprises the greatest conformational difference between the predicted D3R:**1** structure and the D3R:**2** X-ray crystal structure.”

should read:

“Aside from the different rotamer states in the tyrosine toggle switch, the formation of the aromatic cryptic pocket between helices V and VI comprises the greatest conformational difference between the predicted D3R:**1R** structure and the D3R:**2** X-ray crystal structure.”

“The pose identified for **1** based on this extensive simulation data provides a rationale for the potency loss observed with the I183^ECL2^F and V189^5.39^A mutations in the OBS.”

should read:

“The pose identified for **1R** based on this extensive simulation data provides a rationale for the potency loss observed with the I183^ECL2^F and V189^5.39^A mutations in the OBS.”

A revised Supplementary Information file accompanies this correction.

## Supplementary information

Supplementary information

