# Supplementary material for: Dopamine D3 receptor antagonist reveals a cryptic pocket in aminergic GPCRs
Source: Sci Rep. 2018 Jan 17;8:897. doi: 10.1038/s41598-018-19345-7 (PMC5772633; doi:10.1038/s41598-018-19345-7)
Supplement: Supplementary file 1 — Supplementary Information [file 41598_2018_19345_MOESM1_ESM.doc]

Supporting information for:

Dopamine D3 receptor antagonist reveals a cryptic pocket in aminergic GPCRs

Noelia Ferruz,^†,Ϯ^ Stefan Doerr,^†,Ϯ^ Michelle A. Vanase-Frawley,^ξ^ Yaozhong Zou,^ξ^ Xiaomin Chen,^ξ^ Eric S. Marr,^ξ^ Robin T. Nelson,^ξ^ Bethany L. Kormos,^§^ Travis T. Wager,^§^ Xinjun Hou,^§^ Anabella Villalobos,^§^ Simone Sciabola^§^, * and Gianni De Fabritiis ^†,¥,Ϯ^,*

^†^Computational Biophysics Laboratory (GRIB-IMIM), Universitat Pompeu Fabra, Barcelona Biomedical Research Park (PRBB), Doctor Aiguader 88, 08003 Barcelona, Spain

ϮAcellera, PRBB, Doctor Aiguader 88, 08003 Barcelona, Spain

^¥^Institució Catalana de Recerca i Estudis Avançats (ICREA), Passeig Lluis Companys 23, 08010 Barcelona, Spain

^§^Pfizer Worldwide Research and Development, 1 Portland Street, Cambridge, Massachusetts 02139, United States

^ξ^Pfizer Worldwide Research and Development, Eastern Point Road, Groton, Connecticut 06340, United States

*Corresponding author: simone.sciabola@pfizer.com and gianni.defabritiis@upf.edu

This section includes:

Texts S1-S3, Figures S1-S4, Tables S1-S3, and Movie S1.

**SUPPORTING INFORMATION TEXT**

**TEXT S1. Eticlopride/D3R MD simulations.** We performed a batch of 15000 trajectories of 25 ns length for the D3R:**2** system. The systems were built as described in the section **Methods**, with the simulations also running via an adaptive sampling method. We computed the root-mean squared deviation (RMSD) against the X-ray structure for every trajectory and observed that 31 trajectories contained at least one frame within 2.5 Å RMSD (**Fig. S2**), and thus we assumed this methodology was suitable for binding pose prediction on this system. Due to the short size of the trajectories, we could not build a convergent MSM.

**TEXT S2. Adaptive sampling protocol.** We ran the simulations using an unsupervised adaptive sampling protocol.^1^ Three independent batches of a maximum of 125 epochs were run in an initial exploratory phase, each epoch having a maximum of 50 replicas of 25 ns each. We then gathered and analyzed these provisional data (259 μs), which provided an OBS-bound pose but underestimated the timescale of the process, possibly due to short length of the simulations. We selected 250 frames of the most probable binding mode, independently analyzing batches 2 and 3, which together with 250 random poses of the ligand in bulk, comprised the set of 500 input poses for each of the two batches of the second, long set. These two new long batches were then run with trajectories of 100ns each. The protein partially opened between helices 6 and 7, probably due to the shortcomings of the forcefield when running long aggregate simulation times. The absence of cholesterol in the lipid bilayer could also contribute to this effect.^2^ In order to assess the protein conformation, we performed an RMSD analysis of each trajectory compared to the input structure (3PBL) for all of the alpha carbons in the receptor. 1049 trajectories contained a frame with an RMSD over 4 Å (25%), 319 were over 4.5 (8%), 53 were higher than 5 Å (1%) and 10 were over 5.5 Å. Because of the adaptive sampling, these high-RMSD simulations appear in the last epochs; therefore, for the analysis, the first seven epochs on each batch of data were analyzed, totaling 220 μs.

**TEXT S3. Full wild type template D3R sequence**.

**HA signal sequence,** Flag tag, **T4L insertion, His tag**

**MKTIIALSYIFCLVFADYKDDDD**GAPASLSQLSSHLNYTCGAENSTGASQARPHAYYALSYCALILAIVFGNGLVCMAVLKERALQTTTNYLVVSLAVADLLVATLVMPWVVYLEVTGGVWNFSRICCDVFVTLDVMMCTASILNLCAISIDRYTAVVMPVHYQHGTGQSSCRRVALMITAVWVLAFAVSCPLLFGFNTTGDPTVCSISNPDFVIYSSVVSFYLPFGVTVLVYARIYVVLKQRRRK**NIFEMLRIDEGLRLKIYKDTEGYYTIGIGHLLTKSPSLNAAKSELDKAIGRNTNGVITKDEAEKLFNQDVDAAVRGILRNAKLKPVYDSLDAVRRAALINMVFQMGETGVAGFTNSLRMLQQKRWDEAAVNLAKSRWYNQTPNRAKRVITTFRTGTWDAY**GVPLREKKATQMVAIVLGAFIVCWLPFFLTHVLNTHCQTCHVSPELYSATTWLGYVNSALNPVIYTTFNIEFRKAFLKILSCGRPLEVLFQGP**HHHHHHHHHH**.

**SUPPORTING INFORMATION FIGURES**

**Figure S1: Fold-shifts (ki_mut/ki_wt) for each of the 12 mutations for compounds 1-4.**


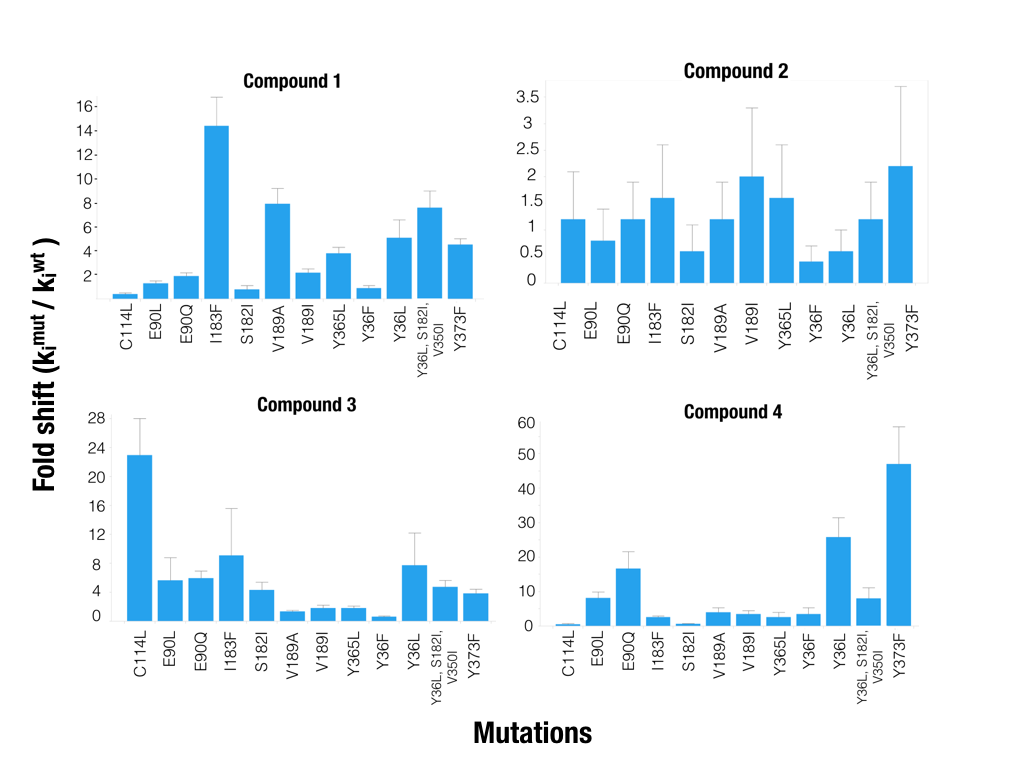


**Figure S2: Summary of the D3R:2 binding simulations. (a)** Superposition of the predicted pose (green) vs. the D3R:**2** X-ray structure (white). The picture depicts the pose that showed the lowest RMSD for protein backbone and ligand heavy atoms (1.8 Å). **(b)** 31 of the trajectories present a frame with an RMSD of 2.5 Å or below. An adaptive protocol was run and thus some simulations’ starting points are at pre-bound conformations.

**
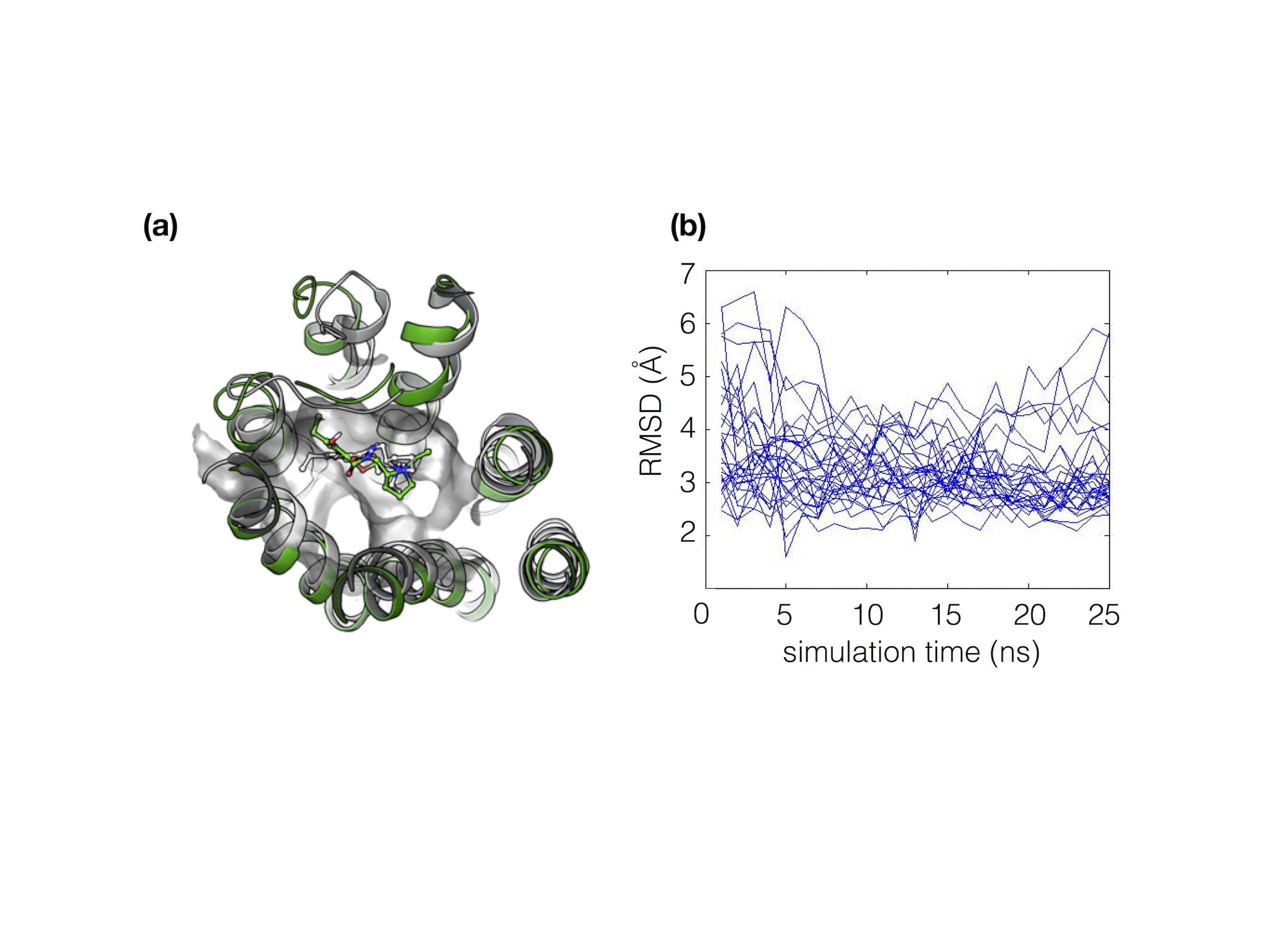
**

**Figure S3: Adaptive sampling protocol for the binding of compound 1 to D3R.**

**(a)** The simulation data was analyzed at different checkpoints, producing bound-like poses in the analysis. **(b)** Evolution of the preferred binding mode from the exploratory set (pose 4) to the long final set (bound pose).


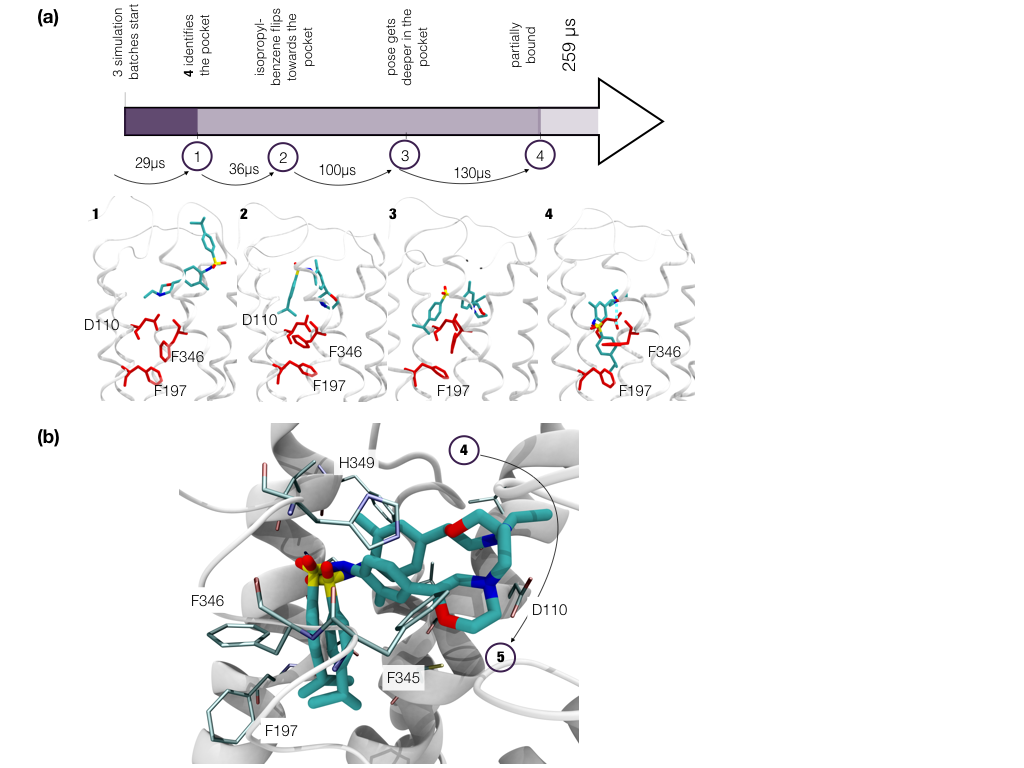


**Figure S4: The eight torsional parameters for the parameterization of compound 1**. QM prediction (black), original cgenff (green), fitted terms used in the simulations (red). The four atoms involved in each dihedral are depicted by red spheres.

**
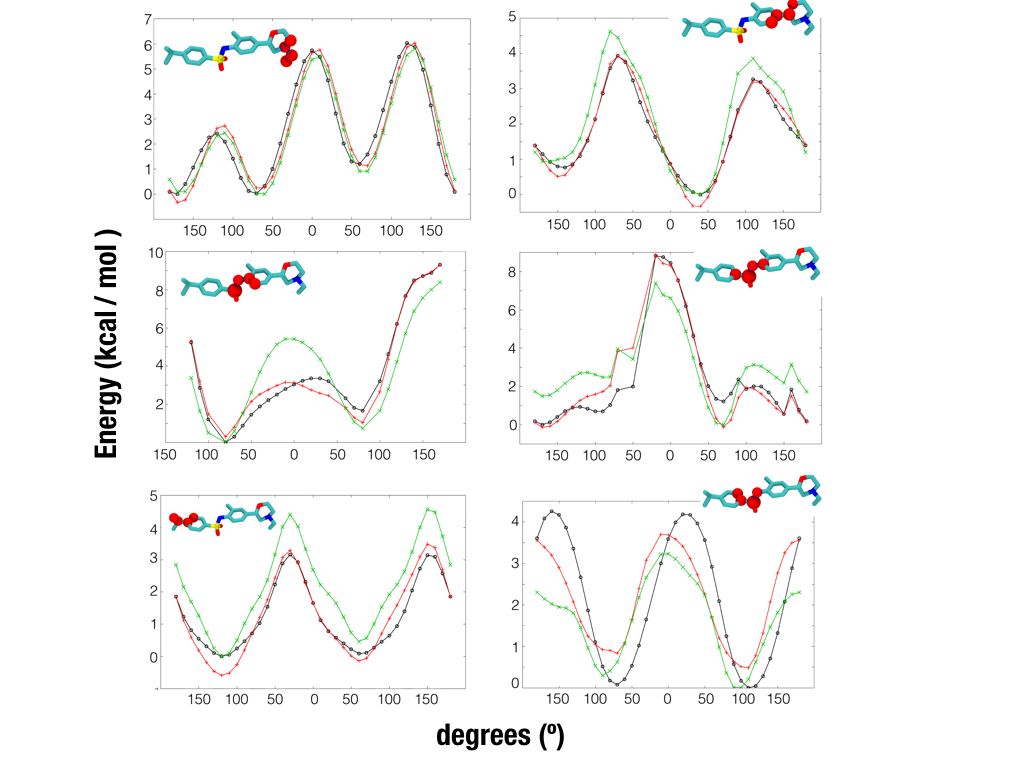
**

**SUPPORTING INFORMATION TABLES**

**Table S1: Affinity measurements for compounds 1 – 4.** k_i_ values for the 12 D3R mutants. Two replicas were measured per compound.

^a^ triple = Y36L, S182I, V350I

| **k_i_ (nM)** | **1** | | **2** | | **3** | | **4** | |
| --- | --- | --- | --- | --- | --- | --- | --- | --- |
| **wt** | 3.4 ± 0.4 | | <0.5 ± 0.3^a^ | | 6.5 ± 1.0 | | 2.5 ± 0.4 | |
| **S182I** | 3.6 | 2.1 | <0.5 | 0.2 | 34.1 | 21.8 | 0.8 | 1.9 |
| **E90L** | 4.9 | 3.7 | <0.6 | <0.2 | 56.6 | 16.9 | 17.5 | 23.1 |
| **Y373F** | 14.8 | 15.8 | 0.7 | 1.4 | 26.7 | 23.0 | 136.8 | 98.4 |
| **E90Q** | 6.0 | 5.4 | 0.6 | 0.5 | 43.6 | 36.5 | 25.0 | 30.9 |
| **V189I** | 8.2 | 6.5 | 0.8 | 1.2 | 14.2 | 9.8 | 10.7 | 6.3 |
| **C114L** | 1.6 | 1.3 | <0.9 | <0.3 | 125.0 | 172.7 | 0.9 | 1.6 |
| **Y365L** | 13.1 | 12.5 | 0.7 | 1.0 | 10.9 | 12.1 | 9.8 | 3.5 |
| **triple**^a^ | 29.5 | 22.0 | 0.5 | 0.7 | 34.0 | 26.7 | 27.0 | 12.8 |
| **Y36L** | 22.0 | 13.0 | <0.2 | 0.4 | 78.0 | 22.0 | 74.0 | 55.0 |
| **I183F** | 55.0 | 43.0 | <0.9 | 0.6 | 100.0 | 18.0 | 6.6 | - |
| **V189A** | 24.0 | 30.0 | 0.5 | 0.7 | 8.1 | 8.5 | 4.0 | 3.6 |
| **Y36F** | 3.2 | 2.5 | 0.3 | <0.2 | 4.0 | 3.6 | 13.0 | 4.5 |

**Table S2: Structural alignment of aminergic GPCRs.** List of X-ray structures of aminergic receptors used for the alignment against the predicted bound pose of compound **1** and calculation of dihedral angles. All of the structures contain a phenylalanine residue at position 6.52.

| **Protein** | **PDB code** | **Resolution** | **Chi1** | **Chi2** |
| --- | --- | --- | --- | --- |
| 5HT_1b_ | 4IAQ | 2.8 | -74.8 | 100.0 |
| 5HT_1b_ | 4IAR | 2.7 | -74.2 | 98.8 |
| 5HT_2b_ | 4IB4 | 2.7 | -71.7 | 106.8 |
| 5HT_2b_ | 4NC3 | 2.8 | -70.9 | 106.2 |
| β1A | 2VT4 | 2.7 | -77.9 | 99.2 |
| β1 | 2Y00 | 2.5 | -73.8 | 103.6 |
| β1A | 2Y01 | 2.6 | -75.4 | 100.9 |
| β1A | 2Y02 | 2.6 | -68.9 | 102.8 |
| β1A | 2Y03 | 2.9 | -77.0 | 103.0 |
| β1A | 2Y04 | 3.1 | -75.2 | 102.6 |
| β1A | 2YCW | 3.0 | -77.7 | 104.0 |
| β1A | 2YCX | 3.3 | -82.8 | 86.9 |
| β1A | 2YCY | 3.2 | -76.9 | 106.4 |
| β1A | 2YCZ | 3.7 | -76.9 | 106.4 |
| β1A | 3ZPQ | 2.8 | -78.9 | 106.4 |
| β1A | 3ZPR | 2.7 | -77.8 | 106.1 |
| β1A | 4AMI | 3.2 | -80.5 | 94.7 |
| β1A | 4AMJ | 2.3 | -77.9 | 99.4 |
| β1A | 4BVN | 2.1 | -75.3 | 96.3 |
| β1A | 4GPO | 3.5 | -85.4 | 101.2 |
| β1A | 5A8E | 2.4 | -80.3 | 98.6 |
| β2A | 2R4R | 3.4 | -75.2 | 70.2 |
| β2A | 2R4S | 3.4 | -76.3 | 72.1 |
| β2A | 2RH1 | 2.4 | -77.7 | 96.8 |
| β2A | 3D4S | 2.8 | -76.6 | 94.1 |
| β2A | 3KJ6 | 3.4 | -79.7 | 86.7 |
| β2A | 3NY8 | 2.8 | -88.9 | 93.5 |
| β2A | 3NY9 | 2.8 | -86.0 | 97.5 |
| β2A | 3NYA | 3.2 | -85.5 | 97.2 |
| β2A | 3P0G | 3.5 | -81.3 | 96.7 |
| β2A | 3PDS | 3.5 | -78.9 | 96.1 |
| β2A | 3SN6 | 3.2 | -90.8 | 83.5 |
| β2A | 4GBR | 4.0 | -80.2 | 91.3 |
| β2A | 4LDE | 2.8 | -80.8 | 96.9 |
| β2A | 4LDL | 3.1 | -78.3 | 91.0 |
| β2A | 4LDO | 3.2 | -77.0 | 94.9 |
| β2A | 4QKX | 3.3 | -74.3 | 102.7 |
| D3R | 3PBL | 2.9 | -82.5 | 82.3 |
| His1 | 3RZE | 3.1 | -84.3 | 94.4 |

**Table S3: List of mutagenesis primers used**. Triple mutants were produced by combining the primers listed below.

| **Mutation** | **Forward primer (5′ → 3′)** | **Reverse primer (5′ → 3′)** |
| --- | --- | --- |
| **C114F** | CGTGACTCTCGATGTGATGATGCTCACCGCGTCAATTTTGAACTTGTGC | GCACAAGTTCAAAATTGACGCGGTGAGCATCATCACATCGAGAGTCACG |
| **E90L** | CCTTGGGTTGTGTACCTGTTGGTAACGGGCGGAGTTTGG | CCAAACTCCGCCCGTTACCAACAGGTACACAACCCAAGG |
| **E90Q** | CTTGGGTTGTGTACCTGCAGGTAACGGGCGGAGTTTGG | CCAAACTCCGCCCGTTACCTGCAGGTACACAACCCAAG |
| **I183F** | CCTACCGTTTGCTCATTCTCAAACCCGGACTTCG | CGAAGTCCGGGTTTGAGAATGAGCAAACGGTAGG |
| **S182I** | CTGGAGATCCTACCGTTTGCATCATATCAAACCCGGACTTCG | CGAAGTCCGGGTTTGATATGATGCAAACGGTAGGATCTCCAG |
| **V189A** | GCTCATTCTCAAACCCGGACTTCGCTATCTACTCCTCTGTTGTGTCG | CGACACAACAGAGGAGTAGATAGCGAAGTCCGGGTTTGAGAATGAGC |
| **V189I** | GCTCAATATCAAACCCGGACTTCATTATCTACTCCTCTGTTGTGTCG | CGACACAACAGAGGAGTAGATAATGAAGTCCGGGTTTGATATTGAGC |
| **Y365L** | CGTCAGTCCGGAATTGCTTTCTGCTACTACTTGG | CCAAGTAGTAGCAGAAAGCAATTCCGGACTGACG |
| **Y36F** | CCTACTACGCGCTGAGTTTCTGCGCGTTGATCCTCG | CGAGGATCAACGCGCAGAAACTCAGCGCGTAGTAGG |
| **Y36L** | CCTACTACGCGCTGAGTCTCTGCGCGTTGATCCTCG | CGAGGATCAACGCGCAGAGACTCAGCGCGTAGTAGG |
| **V350I** | GCCGTTCTTCCTCACCCACATTCTGAATACCCACTGCCAAACC | GGTTTGGCAGTGGGTATTCAGAATGTGGGTGAGGAAGAACGGC |
| **Y373F** | GCTACTACTTGGCTCGGCTTCGTCAATAGTGCTCTCAACC | GGTTGAGAGCACTATTGACGAAGCCGAGCCAAGTAGTAGC |

**SUPPORTING INFORMATION MOVIES**

**Movie S1: Example binding event.** A frame at the bound macrostate was randomly selected and the simulations were traced back through the adaptive epochs. The binding event takes 1.2 μs of simulation time. Residues Phe197^5.47^ and Phe346^6.52^ are shown for reference.

**REFERENCES**

1. Doerr, S. & De Fabritiis, G. On-the-Fly Learning and Sampling of Ligand Binding by High-Throughput Molecular Simulations. *J. Chem. Theory Comput.* **10,** 2064–2069 (2014).

2. Hofsäß, C., Lindahl, E. & Edholm, O. Molecular Dynamics Simulations of Phospholipid Bilayers with Cholesterol. *Biophys. J.* **84,** 2192–2206 (2003).
